# Lifetime Predictions for High-Density Polyethylene under Creep: Experiments and Modeling

**DOI:** 10.3390/polym15020334

**Published:** 2023-01-09

**Authors:** A. D. Drozdov, R. Høj Jermiin, J. de Claville Christiansen

**Affiliations:** Department of Materials and Production, Aalborg University, Fibigerstraede 16, 9220 Aalborg, Denmark

**Keywords:** high-density polyethylene, creep failure, creep endurance limit, lifetime prediction, constitutive modeling

## Abstract

Observations are reported in uniaxial tensile tests with various strain rates, tensile relaxation tests with various strains, and tensile creep tests with various stresses on high-density polyethylene (HDPE) at room temperature. Constitutive equations are developed for the viscoelastoplastic response of semicrystalline polymers. The model involves seven material parameters. Four of them are found by fitting observations in relaxation tests, while the others are determined by matching experimental creep curves. The predictive ability of the model is confirmed by comparing observations in independent short- and medium-term creep tests (with the duration up to several days) with the results of numerical analysis. The governing relations are applied to evaluate the lifetime of HDPE under creep conditions. An advantage of the proposed approach is that it predicts the stress-time-to-failure diagrams with account for the creep endurance limit.

## 1. Introduction

Widespread industrial applications of polymers and polymer composites require an adequate description of their mechanical behavior and failure under constant, monotonic, and periodic loadings. The lifetime assessment and predictions of the long-term response of these materials under creep and fatigue conditions have attracted noticeable attention in the past few decades [1,2,3,4]. These issues are of essential importance for polyethylene pipes, whose failure is driven by the viscoplastic flow and damage accumulation in both the amorphous and crystalline phases [5,6,7].

Although the problem under consideration can be easily formulated, namely, to predict the lifetime of high-density polyethylene (HDPE) under a specific loading program on the basis of experimental data in short-term tests, its solution has not yet been obtained despite of numerous attempts [8,9,10,11,12,13,14,15,16,17,18,19,20,21,22]. This may be explained by the fact that an accurate prediction of the long-term behavior of semicrystalline polymers requires constitutive models that take into account the viscoelastic and viscoplastic responses, and damage accumulation in the crystalline and amorphous phases, and at their boundaries. Such models involve a large number of adjustable parameters that are strongly affected by the microstructure of polymer networks (molecular weight, degree of branching of chains, degree of crystallinity, lamellar thickness, etc.). As the accuracy of determination of these parameters in short-term tests is limited, and the coefficients under consideration evolve slowly with time (due to damage accumulation [23] and the chemical aging [24] of polymers), it is difficult to expect accurate lifetime predictions within the time intervals of tens of years.

The objective of this study is fourfold:

1. To develop a simple model for the viscoelastoplastic response of semicrystalline polymers that involves only seven material parameters. Four of them characterize the viscoelastic behavior, and the remaining three reflect the viscoplastic response.
2. To perform short-term tests on HDPE samples at room temperature (uniaxial tension up to breakage of samples with various strain rates, tensile relaxation tests at various strains, and tensile creep tests with various stresses) and to find material parameters by matching the experimental data.
3. To validate the model by comparing its predictions with observations on HDPE in independent short- and medium-term (up to several days) creep tests.
4. To predict the lifetime of HDPE in long-term creep tests numerically, and to compare these predictions with conventional stress-time-to-failure diagrams.

The novelty of this research consists in the following. A semicrystalline polymer is treated as an equivalent polymer network, whose viscoplastic response is modeled as sliding of junctions between chains with respect to their initial positions. Two mechanisms of sliding are introduced that characterize plastic flows in the crystalline and amorphous regions. The kinetics of flow in the crystallites is described by a modification of the Norton law. To describe the flow in the amorphous phase, a kinetic equation is proposed with the conformable fractional derivative.

To assess the lifetime of polymers under creep conditions, Eyring curves are traditionally applied (they presume a linear dependence between the logarithms of the time to failure $t_{f}$ and the applied stress σ [25]). Two shortcomings of these diagrams are:

(i) They are valid at the first stage of creep only (before changes in microstructure driven by slow growth of microcracks become noticeable [7]).

(ii) They do not account for the creep endurance limit (the stress below which the failure of samples does not occur in creep tests [26]). The presence of this limit is of secondary importance for polymers with large yield stresses $\sigma_{y}$ (because the endurance limit is reached at times exceeding strongly the end-of-life time of polymer structures), but it becomes important for semicrystalline polymers with low $\sigma_{y}$ (noticeable deviations between observations in creep tests on polyethylene samples and the Eyring diagrams are reported in [27,28,29]). The proposed model leads to conventional stress-time-to-failure diagrams at relatively high stresses, and it allows for the accuracy of lifetime predictions to be improved under creep with intermediate stresses when the presence of the endurance limit is to be taken into account.

The exposition is organized as follows. Observations in mechanical tests on HDPE samples are reported in Section 2. A detailed derivation of the model is provided in Section 3. The fitting of the experimental data is performed in Section 4. The model is verified in Section 5 by comparing its predictions with experimental data in independent tests. The results of numerical analysis for long-term creep tests are discussed in Section 4.3. Concluding remarks are formulated in Section 5.

## 2. Materials and Methods

Mechanical tests were conducted on HDPE Borsafe HE3490-LS (density 959 kg/m^3^, melt flow rate 0.25 g/10 min, melting temperature 130 °C) supplied by Borealis. This material is used in pipe systems for water, gas, and sewage.

Dumbbell-shaped specimens (ISO 527-2-1B) with a total length of 145 mm, gauge length of 65 mm, and cross-sectional area of 9.81 mm × 3.95 mm were molded by using injection-moulding machine Ferromatik Milacron K110.

The degree of the crystallinity of specimens was measured by means of a Q1000 differential scanning calorimetry (DSC) apparatus (TA Instruments). Three samples with masses of approximately 8 mg cut from the middle of specimens were heated from 25 to 210 °C with a rate of 10 K/min, and cooled with a rate of 5 K/min down to 25 °C (three cycles of heating–cooling) under nitrogen flow. We found the degree of crystallinity of 55% by dividing the enthalpy of melting ΔH measured in the tests by the enthalpy of melting ΔH=293 J/g of the crystalline polyethylene.

Uniaxial tensile tests were performed by means of testing machine Instron 5568 equipped with an extensometer and a 5 kN load cell. Each test was repeated at least by twice.

Three series of tests were performed: (i) Tensile tests with cross-head speeds $\dot{d}$ ranging from 10 to 100 mm/min up to breakage of samples. (ii) Short-term stress relaxation tests (with a duration of 30 min) with various strains ϵ ranging from 0.01 and 0.05. (iii) Short- and medium-term creep tests (with durations ranging from 15 min to 120 h) with various stresses σ in the interval between 9 and 22 MPa. All experiments were conducted at room temperature (T=21 °C) in a climate-controlled room.

### 2.1. Tensile Tests

**Figure 1:**
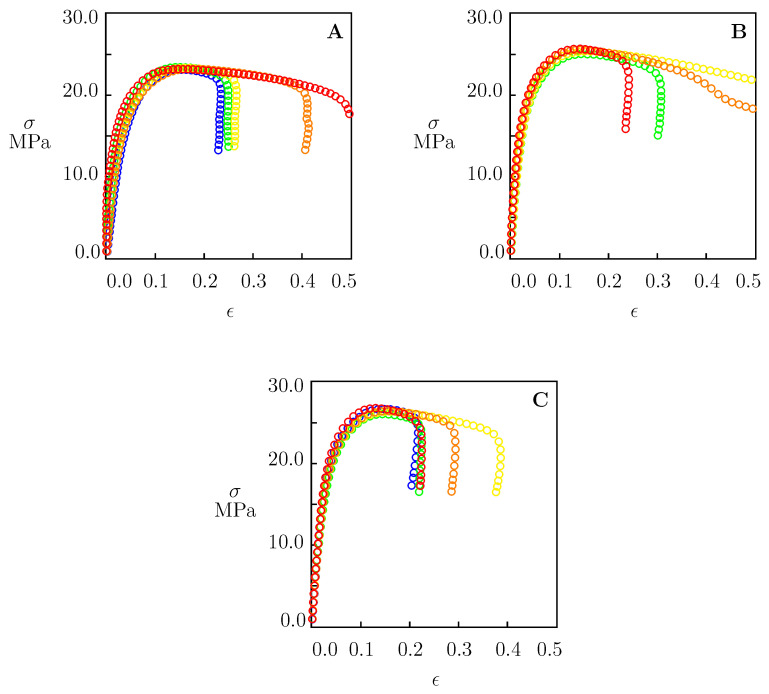
Tensile stress σ versus tensile strain ϵ. Circles: experimental data in tensile tests with cross-head speeds (**A**) 10, (**B**) 50, and (**C**) 100 mm/min.

Uniaxial tensile tests were performed with cross-head speeds $\dot{d}=10$, 50 and 100 mm/min (which corresponded to the strain rates $\dot{\epsilon }=0.002$, 0.01 and 0.02 s^−1^) up to the breakage of the specimens. The cross-head speeds under consideration are conventionally used for the quality assessment of HDPE pipes under quasistatic loading [30,31]. Each test was repeated five times on different samples prepared by injection molding under the same conditions. Experimental data are depicted in Figure 1, where engineering stress σ is plotted versus engineering strain ϵ.

Figure 1 shows that stress σ increased with strain ϵ below the yield point $\epsilon_{y}$, reached its maximum at $\epsilon_{y}\approx 0.15$ (this value is weakly affected by the strain rate), and decreased slightly afterwards. Yield stress $\sigma_{y}$ grew modestly with the strain rate: from 23.2 MPa at $\dot{\epsilon }=0.002$ s^−1^ up to 26.4 MPa at $\dot{\epsilon }=0.02$ s^−1^ (by 13.8%). This growth may be explained by a less pronounced relaxation of tensile stresses (due to a decrease in the duration of loading) and the slowing down of plastic flow in the amorphous phase of HDPE. Figure 1 demonstrates the rather high accuracy of the measurements. For example, the standard deviations of tensile stresses at the yield point read $S_{y}=0.14$ MPa at $\dot{\epsilon }=0.002$ s^−1^ and $S_{y}=0.26$ MPa at $\dot{\epsilon }=0.02$ s^−1^, which means that they remained below 1% of the mean values. However, the strain at break $\epsilon_{b}$ varied in a wide interval between 0.2 and 0.5 at all strain rates $\dot{\epsilon }$.

### 2.2. Relaxation Tests

Short-term stress relaxation tests were performed at strains $\epsilon_{0}=0.01$, 0.02, and 0.05. Each test was repeated twice on different specimens. A specimen was stretched up to the required strain $\epsilon_{0}$ with a cross-head speed of 50 mm/min. Then, the strain was fixed, and the decay in stress σ was measured as a function of relaxation time $t_{\mathrm{rel}}=t-t_{0}$, where $t_{0}$ stands for the instant when the strain ϵ reached the value $\epsilon_{0}$.

**Figure 2:**
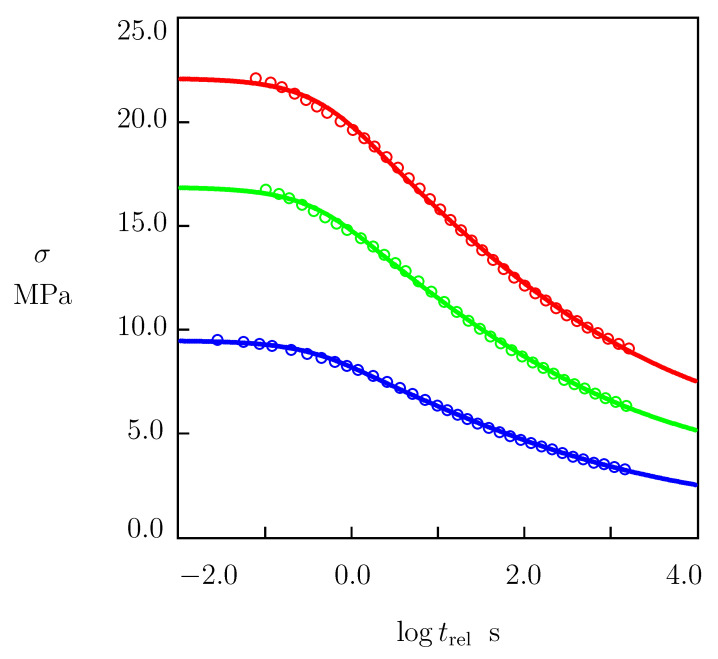
Tensile stress σ versus relaxation time $t_{\mathrm{rel}}$. Circles: experimental data in relaxation tests with various strains ϵ (ϵ=0.01—blue, ϵ=0.02—green, ϵ=0.05—red). Solid lines: results of simulation.

Experimental data in relaxation tests are reported in Figure 2 together with their fits by the model. In this figure, engineering stress σ is plotted versus the logarithm ($\log=\log_{10}$) of relaxation time $t_{\mathrm{rel}}$. Circles stand for the mean values of tensile stress. For each strain $\epsilon_{0}$ under consideration, deviations between the stress measured in a test and the mean stress depicted in Figure 2 did not exceed 2%. Solid lines denote results of numerical simulation described in detail in Section 4.1.

### 2.3. Creep Tests

Three series of uniaxial creep tests were performed with tensile stresses σ=9, 11, 12, 13, 14, 14.5, 15, 17.5, 18, 19, 20, and 20.5 MPa.

The first series of creep tests was performed with stresses 9, 11, 13, 14, 15, 17.5 and 20.5 MPa. The tests with stresses σ=9, 11, 13, 14, and 15 MPa were conducted with a constant duration of 4 h. Creep tests with stresses σ=17.5 and 20.5 MPa were performed up to the breakage of the samples. Each test was repeated by twice on different specimens. First, a specimen was stretched up to the required stress σ with a cross-head speed of 50 mm/min. Afterwards, the stress was fixed, and an increase in tensile strain ϵ was measured as a function of creep time $t_{\mathrm{cr}}=t-t_{0}$, where $t_{0}$ stands for the instant when the required stress σ is reached.

**Figure 3:**
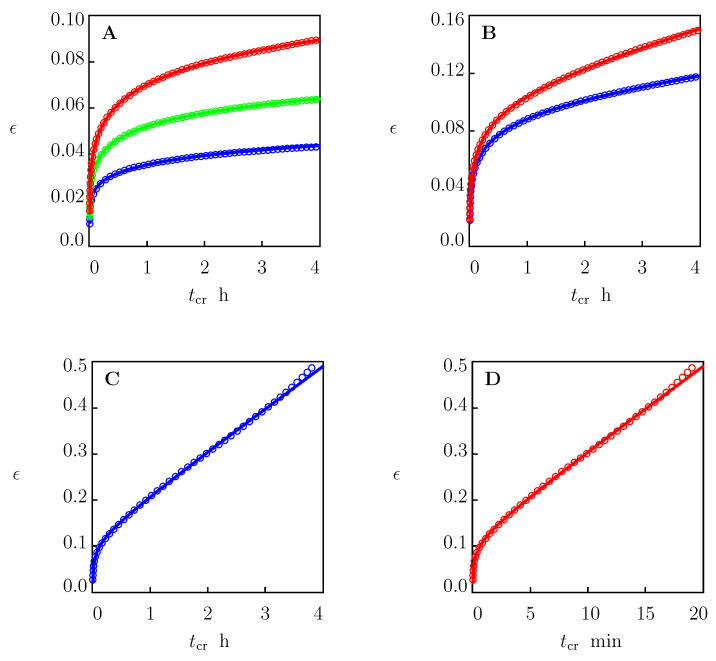
Tensile strain ϵ versus creep time $t_{\mathrm{cr}}$. Circles: experimental data in creep tests with various tensile stresses σ. Solid lines: results of numerical analysis. (**A**)—σ=9.0 (blue), σ=11 (green), σ=13 (red), (**B**)—σ=14.0 (blue), σ=15 (red), (**c**)—σ=17.5 (blue), (**D**)—σ=20.5 (red) MPa.

The experimental data are reported in Figure 3 with the results of numerical analysis. In this figure, tensile strain ϵ is plotted versus creep time $t_{\mathrm{cr}}$. Circles denote the mean values of tensile strain. For each stress σ under investigation, deviations between the strain measured in a test and the mean strain presented in Figure 3 did not exceed 2% for σ below 14 MPa and 3% for higher stresses. Solid lines stand for the results of simulation discussed in Section 4.1.

## 3. Constitutive Model

To develop a model for the viscoelastoplastic response and creep failure of HDPE that involved a relatively small number of material parameters, the following assumptions are introduced.

Focusing on experimental data in uniaxial tests, we disregarded volume deformation and treated HDPE as an incompressible material. This assumption is in accordance with the observations [17,32] showing that the Poisson ratio of polyethylene ν belongs to the interval between 0.48 and 0.49.

To simplify the derivation of the constitutive equations, we confined ourselves to the analysis of isothermal response at small strains. The neglect of geometrical nonlinearity seems to be acceptable for samples broken at tensile strains close to 0.2 (Figure 1). It can, however, lead to some inaccuracy when fracture occurs at higher strains (up to 0.5). We will show in what follows that our lifetime predictions are weakly affected by this inaccuracy.

HDPE is a semicrystalline polymer containing two phases (amorphous and crystalline). Following [14], the presence of interphases and the rigid amorphous phase (chains with reduced molecular mobility located between lamellar) was disregarded. The viscoplastic deformation of the amorphous phase reflects (i) chain slip through the crystals, (ii) the sliding of tie chains along and their detachment from lamellar blocks, and (iii) the detachment of chain folds and loops from the surfaces of crystal blocks [33]. The viscoplastic deformation in the crystalline phase reflects (i) interlamellar separation, (ii) the rotation and twist of lamellae, and (iii) the fine (homogeneous shear of layerlike crystalline structures) and (iv) coarse (heterogeneous interlamellar sliding) slip of lamellar blocks [34]. The viscoelastic response of HDPE is associated with (i) the relaxation of stresses in chains located in the amorphous regions and (ii) time-dependent decay in forces transmitted to the crystalline skeleton by tie chains [35,36].

A detailed account of the evolution of the microstructure of semicrystalline polymers under loading leads to a strong increase in the number of parameters to be found by matching observations [22,37]. To make the model tractable, we treated a semicrystalline polymer as an equivalent nonaffine network of polymer chains connected by permanent and temporary junctions [38]. Nonaffinity means that junctions can slide with respect to their reference positions under deformation. As the sliding process reflects the viscoplastic flows in the amorphous and crystalline phases, the strain tensor for plastic deformation $\epsilon_{p}$ is presumed to consist of two components,

(1) $$\epsilon_{p}=\epsilon_{\mathrm{pa}}+\epsilon_{\mathrm{pc}},$$

which describe flows in the bulk amorphous phase ($\epsilon_{\mathrm{pa}}$) and the crystalline skeleton ($\epsilon_{\mathrm{pc}}$), respectively. A similar decomposition of the plastic strain into two components was introduced in [39] for the analysis of viscoplastic deformations in semicrystalline polymers at finite strains.

We supposed that (i) tensors $\epsilon_{\mathrm{pa}}$ and $\epsilon_{\mathrm{pc}}$ were traceless (volumetric plastic deformation is neglected), and (ii) their evolution was governed by the following equations:

(2) $${\dot{\epsilon }}_{\mathrm{pa}}=\sqrt{\frac{3}{2}}\frac{s}{s_{\mathrm{eq}}}\varphi_{a}\left(t,s_{\mathrm{eq}}\right),\;{\dot{\epsilon }}_{\mathrm{pc}}=\sqrt{\frac{3}{2}}\frac{s}{s_{\mathrm{eq}}}\varphi_{c}\left(s_{\mathrm{eq}}\right).$$

Here, the superscript dot denotes the derivative with respect to time *t*, s stands for the deviatoric component of stress tensor σ,

(3) $$s_{\mathrm{eq}}=\sqrt{s:s}$$

is the equivalent stress, and the colon denotes convolution. Analytical expressions for the non-negative scalar functions $\varphi_{a}\left(t,s_{\mathrm{eq}}\right)$ and $\varphi_{c}\left(s_{\mathrm{eq}}\right)$ are introduced in what follows. An analogous treatment of the viscoplastic strain (as a sum of two components obeying time-dependent and time-independent kinetic equations) was suggested in [40].

Chains in the equivalent network are bridged by permanent and temporary bonds. Both ends of a permanent chain are merged with the network by permanent cross-links. At least one end of a temporary chain is connected with the network by a temporary (physical) bond that can break (dissociate) and reform (reassociate). When both ends of a temporary chain are attached to the network, the chain is in its active state. When an end of an active chain separates from its junction at some instant $\tau_{1}$, the chain is transformed into the dangling state. When the free end of a dangling chain merges with the network at an instant $\tau_{2}>\tau_{1}$, the chain returns into the active state. Attachment and detachment events occur at random times, being driven by thermal fluctuations.

The equivalent polymer network consist of mesoregions with various activation energies for breakage of temporary bonds. The rate of separation of an active chain from its junction in a mesodomain with activation energy *u* is governed by the Eyring equation:

(4) $$\Gamma =\gamma \exp\left(-\frac{u}{k_{B}T}\right),$$

where γ stands for an attempt rate, *T* is the absolute temperature, and $k_{B}$ denotes the Boltzmann constant. For isothermal processes at a fixed temperature *T*, we introduce the dimensionless energy $v=u/(k_{B}T)$, and find that

(5) Γ=γexp(−v).

With reference to the random energy model [41], the quasi-Gaussian formula is adopted for the distribution function of mesodomains with various activation energies *v*,

(6) $$f\left(v\right)=f_{0}\exp\left(-\frac{v^{2}}{2\Sigma^{2}}\right)\;\left(v\geq 0\right).$$

The advantage of Equation (6) is that function *f* is characterized by the only parameter Σ>0 that serves as a measure of inhomogeneity of the equivalent network [36]. Prefactor $f_{0}$ is determined from the normalization condition

(7) $$\int_{0}^{\infty }f\left(v\right)dv=1.$$

The rearrangement process in an inhomogeneous polymer network is described by a function n(t,τ,v) that equals the number of temporary chains (per unit volume) at time t≥0 that belong to mesodomains with activation energy *v* and have returned into the active state before instant τ≤t. In particular, the number of temporary chains that were active at the instant t=0 and were not separated from their junctions until time *t* is given by n(t,0,v). The number of active chains in mesodomains with activation energy *v* at time *t* reads $n^{*}\left(t,v\right)=n\left(t,\tau {|}_{\tau =t},v\right)$. The number of temporary chains that were active at the initial instant and detach from their junctions within the interval [t,t+dt] reads −∂n/∂t(t,0,v)dt. The number of dangling chains that return into the active state within the interval [τ,τ+dτ] is given by r(τ,v)dτ with

(8) $$r\left(\tau ,v\right)=\frac{\partial n}{\partial \tau }\left(t,\tau ,v\right){|}_{t=\tau }.$$

The number of temporary chains that merged (for the last time) with the network within the interval [τ,τ+dτ] and detach from their junctions within the interval [t,t+dt] equals $-\partial^{2}n/\partial t\partial \tau \left(t,\tau ,v\right)\;dtd\tau$.

We presumed the number of active chains in mesodomains with activation energy *v* to remain independent of time,

(9) $$n^{*}\left(t,v\right)=N_{a}f\left(v\right),$$

where $N_{a}$ stands for the number of active chains per unit volume.

The separation of temporary chains from their junctions is described by the following kinetic equations:

(10) $$\frac{\partial n}{\partial t}\left(t,0,v\right)=-\Gamma \left(v\right)n\left(t,0,v\right),\;\frac{\partial^{2}n}{\partial t\partial \tau }\left(t,\tau ,v\right)=-\Gamma \left(v\right)\frac{\partial n}{\partial \tau }\left(t,\tau ,v\right),$$

Equation (10) means that the rate of the transformation of active chains into the dangling state is proportional to their number in an appropriate mesoregion. The integration of Equation (10) with conditions (8) and (9) implies that

(11) $$\begin{array}{ccc} n(t,0,v) & = & N_{a}f\left(v\right)\exp\left[-\Gamma \left(v\right)t\right],\\ \frac{\partial n}{\partial \tau }\left(t,\tau ,v\right) & = & N_{a}f\left(v\right)\Gamma \left(v\right)\exp\left[-\Gamma \left(v\right)\left(t-\tau \right)\right]. \end{array}$$

The strain energy of a chain is determined by the following conventional formula:

(12) $$w=\frac{1}{2}\mu \epsilon_{e}:\epsilon_{e},$$

where μ stands for the rigidity of a chain, and the strain tensor for elastic deformations $\epsilon_{e}$ reads

(13) $$\epsilon_{e}=\epsilon -\epsilon_{p},$$

where ϵ is the strain tensor for macrodeformation.

Under the assumption that the energy of interchain interaction is accounted for by the incompressibility condition, the strain energy density of the equivalent network *W* is calculated as the sum of the strain energies of permanent chains and active transient chains:

(14) $$\begin{array}{ccc} W(t) & = & \frac{1}{2}\mu [\left(N_{p}+\int_{0}^{\infty }n\left(t,0,v\right)\;dv\right)\epsilon_{e}\left(t\right):\epsilon_{e}\left(t\right)\\ & & +\int_{0}^{\infty }dv\int_{0}^{t}\frac{\partial n}{\partial \tau }\left(t,\tau ,v\right)\left(\epsilon_{e}\left(t\right)-\epsilon_{e}\left(\tau \right)\right):\left(\epsilon_{e}\left(t\right)-\epsilon_{e}\left(\tau \right)\right)\;d\tau ], \end{array}$$

where $N_{p}$ is the number of permanent chains per unit volume.

The first term in Equation (14) equals the sum of the strain energies of permanent chains and temporary chains that have not been rearranged within the interval [0,t]. The last term expresses the strain energy of chains that have last merged with the network at various instants τ∈[0,t] in mesoregions with various activation energies *v*. We supposed that stresses totally relax in dangling chains before they merge with the network, which implies that the strain energy (at time *t*) of a chain transformed into the active state at time τ depends on the relative elastic strain tensor $\epsilon_{e}^{*}\left(t,\tau \right)=\epsilon_{e}\left(t\right)-\epsilon_{e}\left(\tau \right)$.

Differentiating Equation (14) with respect to time, and using Equations (7), (9) and (10), we find that

(15) $$\frac{dW}{dt}\left(t\right)=\mu \left[\left(N_{p}+N_{a}\right)\epsilon_{e}\left(t\right)-\int_{0}^{\infty }dv\int_{0}^{t}\frac{\partial n}{\partial \tau }\left(t,\tau ,v\right)\epsilon_{e}\left(\tau \right)d\tau \right]:\frac{d\epsilon_{e}}{dt}\left(t\right)-J\left(t\right),$$

where

(16) $$\begin{array}{ccc} J(t) & = & \frac{1}{2}\mu [\int_{0}^{\infty }\Gamma \left(v\right)n\left(t,0,v\right)dv\epsilon_{e}\left(t\right):\epsilon_{e}\left(t\right)\\ & & +\int_{0}^{\infty }\Gamma \left(v\right)dv\int_{0}^{t}\frac{\partial n}{\partial \tau }\left(t,\tau ,v\right)\left(\epsilon_{e}\left(t\right)-\epsilon_{e}\left(\tau \right)\right):\left(\epsilon_{e}\left(t\right)-\epsilon_{e}\left(\tau \right)\right)\;d\tau ]\geq 0. \end{array}$$

Under isothermal the isothermal deformation of an incompressible medium, the Clausius–Duhem inequality reads [10]:

(17) $$Q=-\frac{dW}{dt}+s:\frac{d\epsilon }{dt}\geq 0,$$

where *Q* stands for the internal dissipation per unit volume and unit time. It follows from Equation (15) that Equation (17) is satisfied for an arbitrary deformation program, provided that

(18) $$\sigma =-pI+\mu \left[\left(N_{p}+N_{a}\right)\epsilon_{e}\left(t\right)-\int_{0}^{\infty }dv\int_{0}^{t}\frac{\partial n}{\partial \tau }\left(t,\tau ,v\right)\epsilon_{e}\left(\tau \right)d\tau \right],$$

where *p* stands for an unknown pressure, and I denotes the unit tensor. Inserting Equation (18) into Equation (17), and using Equations (1)–(3) and (16), we find that

(19) $$Q=\sqrt{\frac{3}{2}}s_{\mathrm{eq}}\left[\varphi_{a}\left(t,s_{\mathrm{eq}}\right)+\varphi_{c}\left(s_{\mathrm{eq}}\right)\right]+J\geq 0.$$

Using Equation (11), and introducing the notation

$$G=\frac{\mu }{2}\left(N_{p}+N_{a}\right),\;\kappa =\frac{N_{p}}{N_{p}+N_{a}},$$

we present Equation (18) in the form

(20) $$\sigma \left(t\right)=-p\left(t\right)I+2G\left[\epsilon_{e}\left(t\right)-\kappa \int_{0}^{\infty }\Gamma \left(v\right)f\left(v\right)dv\int_{0}^{t}\exp\left(-\Gamma (v)(t-\tau )\right)\epsilon_{e}\left(\tau \right)d\tau \right].$$

Equations (1), (2), (5), (6), (13) and (20) provide constitutive relations for the viscoelastoplastic response of semicrystalline polymers at small strains. They involve four material parameters that characterize the viscoelastic behavior (*G*, κ, γ and Σ) and two functions ($\varphi_{a}$ and $\varphi_{c}$) that describe the viscoplastic flows in the amorphous and crystalline phases.

Under uniaxial tension, Equation (20) is simplified. The strain tensor for macrodeformation reads

(21) $$\epsilon =\epsilon \left[i_{1}i_{1}-\frac{1}{2}\left(i_{2}i_{2}+i_{3}i_{3}\right)\right],$$

where ϵ=ϵ(t) stands for tensile strain in a specimen, and $i_{k}$ (k=1,2,3) denote unit vectors of a Cartesian frame. Strain tensors $\epsilon_{p}$ and $\epsilon_{e}$ obey Equation (21) with the coefficients $\epsilon_{p}$ and $\epsilon_{e}$, respectively. Inserting Expression (21) into Equation (20), and taking into account that

(22) $$\sigma =\sigma i_{1}i_{1},$$

where σ stands for the tensile stress, we arrive at the following formula:

(23) $$\sigma \left(t\right)=E\left[\epsilon_{e}\left(t\right)-\kappa \int_{0}^{\infty }\Gamma \left(v\right)f\left(v\right)dv\int_{0}^{t}\exp\left(-\Gamma (v)(t-\tau )\right)\epsilon_{e}\left(\tau \right)d\tau \right].$$

Here, E=3G is Young’s modulus,

(24) $$\epsilon_{e}\left(t\right)=\epsilon \left(t\right)-\epsilon_{p}\left(t\right),$$

and $\epsilon_{p}$ is given by Equation (1),

(25) $$\epsilon_{p}=\epsilon_{\mathrm{pa}}+\epsilon_{\mathrm{pc}}.$$

Equation (22) implies that

(26) $$s=\frac{2\sigma }{3}\left[i_{1}i_{1}-\frac{1}{2}\left(i_{2}i_{2}+i_{3}i_{3}\right)\right].$$

Equations (3) and (26) yields

(27) $$s_{\mathrm{eq}}=\sigma \sqrt{\frac{2}{3}}.$$

The insertion of Equations (21), (26) and (27) into Equation (2) results in the kinetic equations for the viscoplastic strains $\epsilon_{\mathrm{pa}}$ and $\epsilon_{\mathrm{pc}}$:

(28) $${\dot{\epsilon }}_{\mathrm{pa}}=\varphi_{a}\left(t,\sigma \sqrt{\frac{2}{3}}\right),\;{\dot{\epsilon }}_{\mathrm{pc}}=\varphi_{c}\left(\sigma \sqrt{\frac{2}{3}}\right).$$

This study focuses on the mechanical response of semicrystalline polymers in relaxation and creep tests whose characteristic times belong to intervals from minutes to hundreds of hours. Disregarding the interval of ramp loading (a few seconds), we set

(29) $$\epsilon_{e}\left(t\right)=\epsilon_{0}-\epsilon_{p0}\;\left(t>0\right),$$

where $\epsilon_{p0}$ stands for the plastic strain at the beginning of the relaxation process. The substitution of Equation (29) into Equation (23) yields

(30) $$\sigma \left(t\right)=\sigma_{0}\left[1-\kappa \int_{0}^{\infty }f\left(v\right)\left(1-\exp(-\Gamma (v)t)\right)dv\right]$$

with

(31) $$\sigma_{0}=E\left(\epsilon_{0}-\epsilon_{p0}\right).$$

It follows from Equation (31) that, at relatively small strains $\epsilon_{0}$ (below a threshold at which the plastic deformation occurs),

(32) $$\sigma_{0}=E\epsilon_{0}.$$

Given a strain $\epsilon_{0}$, Equations (30) and (32), together with Equation (5) for Γ(v) and Equation (6) for f(v) involve four material parameters: (i) Young’s modulus *E*. (ii) The rate of separation of active chains from their junctions γ. (iii) The measure of inhomogeneity of the equivalent network Σ. (iv) The fraction of reversible bonds in the network κ. These quantities are found in Section 4 by matching the experimental data depicted in Figure 2.

To fit the experimental creep diagrams, we disregarded the interval of ramp loading and applied Equation (23) with a fixed tensile stress:

σ(t)=σ(t>0).

Inserting this expression into Equation (23), we find that

(33) $$\epsilon_{e}\left(t\right)=\frac{\sigma }{E}+\kappa Z\left(t\right),$$

where

(34) $$Z\left(t\right)=\int_{0}^{\infty }f\left(v\right)z\left(t,v\right)dv$$

and

$$z\left(t,v\right)=\Gamma \left(v\right)\int_{0}^{t}\exp\left(-\Gamma (v)(t-\tau )\right)\epsilon_{e}\left(\tau \right)d\tau .$$

The differentiation of this relation with respect to time implies that function z(t,v) obeys the following differential equation:

(35) $$\frac{\partial z}{\partial t}=\Gamma \left(v\right)\left(\epsilon_{e}-z\right),\;z\left(0,v\right)=0.$$

Tensile strain ϵ is determined from Equations (24) and (25):

(36) $$\epsilon =\epsilon_{e}+\epsilon_{\mathrm{pa}}+\epsilon_{\mathrm{pc}}.$$

Viscoplastic flow in the crystalline phase is described by the conventional Norton equation (see [1,4] for historical surveys):

(37) $$\epsilon_{\mathrm{pc}}\left(t\right)=Bt$$

with

(38) $$B=0\;\left(\sigma <\sigma_{*}\right),\;B=B_{1}{\left(\sigma -\sigma_{*}\right)}^{n}\;\left(\sigma \geq \sigma_{*}\right).$$

where $B_{1}$ and *n* are adjustable parameters. Equation (38) is modified by the introduction of a threshold stress $\sigma_{*}$, below which the viscoplastic flow in spherulites is not observed (the response of the crystalline skeleton in creep tests with $\sigma <\sigma_{*}$ is presumed to be purely elastic).

Viscoplastic flow in the amorphous phase under creep conditions is described by the following equation:

(39) $$\epsilon_{\mathrm{pa}}\left(t\right)=A\left[1-\exp(-\alpha \sqrt{t})\right]$$

with coefficients *A* and α affected by tensile stress σ. Here, *A* denotes the maximal viscoplastic strain induced by sliding of junctions between chains, and α characterizes the rate of the slippage of junctions with respect to their initial positions. At small times *t*, Equation (39) is transformed into the relation suggested by Bailey [42]:

$$\epsilon_{\mathrm{pa}}\left(t\right)=A\alpha \sqrt{t}.$$

An analog of Equation (39) (with $\sqrt{t}$ replaced with *t*) is conventionally used to describe at the initial stage of flow of dislocations in crystalline materials [43].

Equations (37) and (39) imply that the initial (at the beginning of the creep process) viscoplastic strain $\epsilon_{p0}$ vanishes. This assumption seems reasonable at relatively low stresses $\left(\sigma <\sigma_{*}\right)$. We applied it for arbitrary tensile stresses σ due to the presence of a weak singularity in Equation (39) at t=0: the strain $\epsilon_{\mathrm{pa}}$ grows so rapidly at small *t* that it is not necessary to introduce a nonzero initial condition for this quantity.

For a given tensile stress σ, the corresponding viscoplastic flow under creep conditions is determined by three material parameters: (i) The rate of the sliding of junctions in the amorphous phase with respect to their initial positions α. (ii) The maximal viscoplastic strain induced by sliding of junctions *A*. (iii) The rate of plastic deformation in the crystalline phase *B*.

To characterize the effect of stress σ on the coefficients in Equation (39), we presumed *A* to vanish at low stresses (below $\sigma_{*}$), to grow monotonically with σ at moderate stresses, and to reach its ultimate value $A_{1}$ at relatively high stresses:

(40) $$A=0\;\left(\sigma <\sigma_{*}\right),\;A=A_{1}\left[1-\exp\left(-a{\left(\sigma -\sigma_{*}\right)}^{2}\right)\right]\;\left(\sigma \geq \sigma_{*}\right),$$

where *a* and $A_{1}$ are material constants. Equation (40) is similar to the relations for creep ductility discussed in [44]. Its advantage is that Equation (40) involves a smaller number of material parameters.

To predict the stress-induced acceleration of the sliding of junctions, we adopted the power-law relation for coefficient α:

(41) $$\alpha =\alpha_{0}\;\left(\sigma <\sigma_{*}\right),\;\alpha =\alpha_{0}+\alpha_{1}{\left(\sigma -\sigma_{*}\right)}^{m},$$

where $\alpha_{0}$, $\alpha_{1}$ and *m* are material parameters. Equation (41) implies that slippage of junctions is weakly affected by tensile stresses when they remain relatively small, but the rate of this process grows strongly with stresses at higher σ values.

Although Equations (38), (40) and (41) were suggested for uniaxial tensile creep, they can be extended to an arbitrary three-dimensional deformation by means of Equation (2) with

$$\varphi_{a}=A\left[1-\exp(-\alpha \sqrt{t})\right],\;\varphi_{c}=Bt.$$

The differentiation of these equalities with respect to time implies that

(42) $${\dot{\varphi }}_{a}=\frac{\alpha }{2\sqrt{t}}\left(A-\varphi_{a}\right),\;{\dot{\varphi }}_{c}=B.$$

The last relation in Equation (42) is an ordinary differential equation for function $\varphi_{c}$. The first relation reads

(43) $$D_{\frac{1}{2}}\varphi_{a}=\frac{\alpha }{2}\left(A-\varphi_{a}\right),$$

where

$$D_{\beta }f\left(t\right)=\lim_{\delta \to 0}\frac{f\left(t+\delta t^{1-\beta }\right)-f\left(t\right)}{\delta }$$

is the conformable fractional derivative of function f(t) of order β∈(0,1) [45,46]. An advantage of Equation (43) compared with other models in the viscoplasticity of polymers involving the Riemann–Liouville and Caputo fractional derivatives [47,48] is that its solution can be presented in analytical form with the help of elementary functions.

## 4. Results and Discussion

### 4.1. Fitting of Experimental Data

To find material parameters for HDPE, we fitted the experimental data reported in Figure 2 and Figure 3. We begin with the analysis of observations in relaxation tests (Figure 2). Each set of data was matched separately by means of Equation (30), where Γ(v) is given by Equation (5), f(v) was determined by Equation (6), and $\sigma_{0}$ was found from Equation (32). Given γ and Σ, coefficients $\sigma_{0}$ and κ were calculated by using the least-squares technique. Parameters γ and Σ were determined with the nonlinear regression method from the condition of the minimum for the following expression:

$$\sum_{k}{\left[\sigma_{\exp}\left(t_{k}\right)-\sigma_{\mathrm{sim}}\left(t_{k}\right)\right]}^{2},$$

where summation was performed over all instants $t_{k}$ at which the data are presented, $\sigma_{\exp}$ is the tensile stress measured in a relaxation test, and $\sigma_{\mathrm{sim}}$ is given by Equation (30). Figure 2 demonstrates good agreement between the experimental data and their approximation of the model with the material constants collected in Table 1. 

Table 1 shows that coefficients γ, Σ, and κ were practically independent of tensile strain ϵ. It follows from Equation (32) and Table 1 that Young’s modulus reads E=0.952 GPa.

We proceeded with fitting experimental creep diagrams in Figure 3. Each set of data was matched separately by using the following algorithm. Given a stress σ, viscoelastic strain $\epsilon_{e}$ is determined from Equations (33)–(35). These equations were integrated numerically with the Runge–Kutta method with material parameters *E*, γ, Σ, and κ listed in the first line of Table 1. Afterwards, the viscoplastic strain $\epsilon_{p}=\epsilon_{\mathrm{pa}}+\epsilon_{\mathrm{pc}}$ was calculated from Equation (36). Function $\epsilon_{p}\left(t_{\mathrm{cr}}\right)$ was approximated with the following equation:

(44) $$\epsilon_{p}=A\left[1-\exp(-\alpha \sqrt{t_{c}})\right]+Bt_{c},$$

which follows from Equations (37) and (39). Given α, coefficients *A* and *B* were found with the least-squares technique. Parameter α was determined with the nonlinear regression method from the condition of the minimum for the following expression:

$$\sum_{k}{\left[\epsilon_{p\;\exp}\left(t_{k}\right)-\epsilon_{p\;\mathrm{sim}}\left(t_{k}\right)\right]}^{2},$$

where summation was performed over all instants $t_{k}$ at which the data are reported, $\epsilon_{p\;\exp}$ is determined from Equation (36), and $\epsilon_{p\;\mathrm{sim}}$ is given by Equation (44). After finding coefficients α, *A* and *B*, the experimental creep curve $\epsilon (t_{c})$ was approximated with Equation (36), with $\epsilon_{e}$ given by Equations (33)–(35), and $\epsilon_{\mathrm{pa}}$, $\epsilon_{\mathrm{pc}}$ determined by Equations (37) and (39).

**Figure 4:**
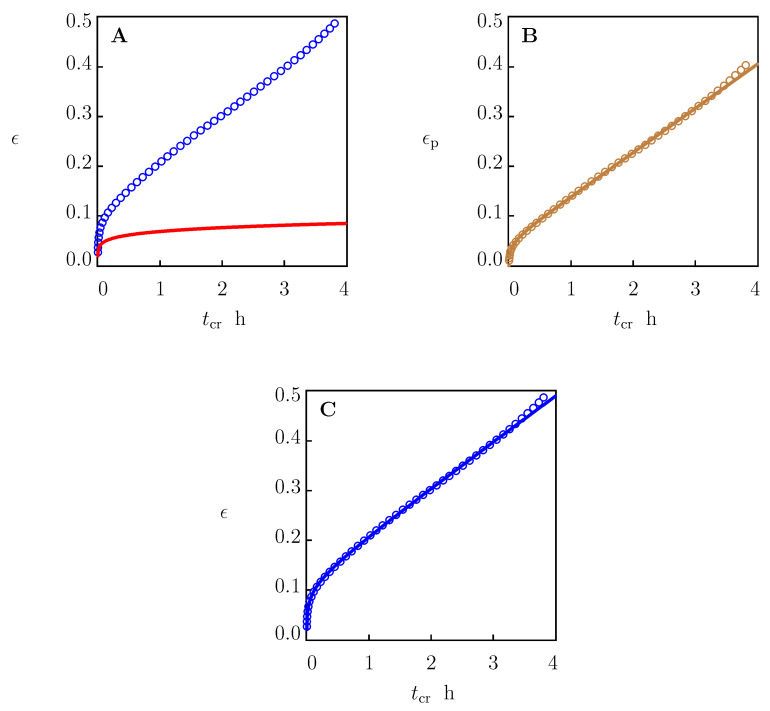
(**A**) Tensile strain ϵ versus creep time $t_{\mathrm{cr}}$. Circles: experimental data in creep tests with tensile stress σ = 17.5 MPa. Solid line: prediction of the model in linear viscoelasticity. (**B**) Plastic strain $\epsilon_{p}$ versus creep time $t_{\mathrm{cr}}$. Circles: treatment of experimental data. Solid line: results of simulation based on the model in viscoplasticity. (**C**) Tensile strain ϵ versus creep time $t_{\mathrm{cr}}$. Circles: experimental data. Solid line: results of simulation based on the model in viscoelastoplasticity.

This procedure is illustrated in Figure 4 for the creep test with σ=17.5 MPa. Observations and the solution of Equations (33)–(35) with the parameters collected in the first line of Table 1 are presented in Figure 4A. The difference between the experimental data (circles) and the linear viscoelastic response of the specimen (solid line) was determined with Equation (24). Function $\epsilon_{p}(t_{c}$) found from Equation (24) is depicted in Figure 4B together with its approximation via Equation (44) with the parameters listed in Table 2. Comparison of the experimental data with results of numerical analysis is shown in Figure 4C.

**Figure 5:**
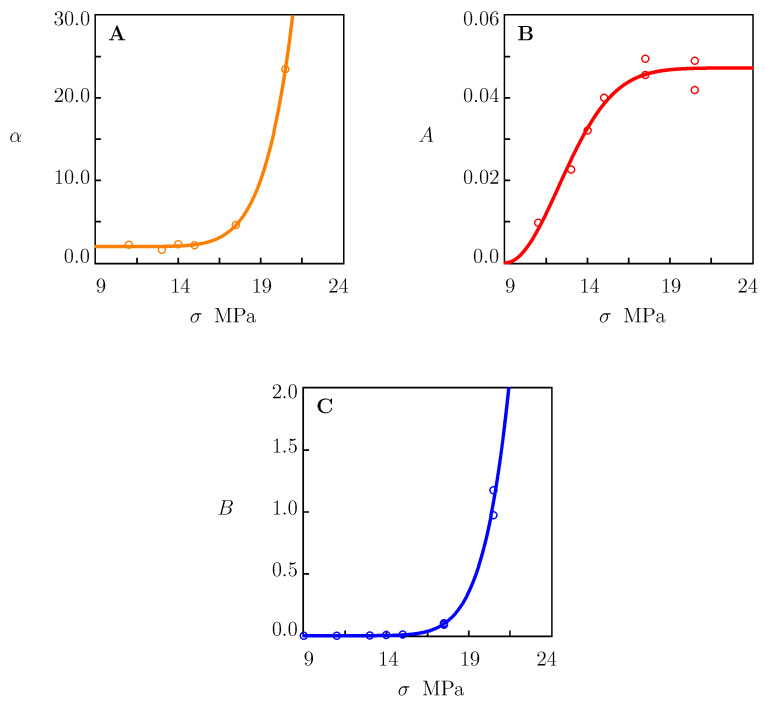
Parameters (**A**) α, (**B**) *A*, and (**C**) *B* versus tensile stress σ. Circles: treatment of experimental data in creep tests. Solid lines: results of numerical analysis.

Quantities α, *A*, and *B* are plotted versus tensile stress σ in Figure 5. The data were approximated with Equations (38), (40) and (41) with $\sigma_{*}=9.0$ MPa and the coefficients reported in Table 2. Stress $\sigma_{*}$ is found from the condition that the response of HDPE in a creep test with σ=9 MPa is purely viscoelastic. This statement is confirmed by Figure 3A, which shows good agreement between the observations in the creep test and the predictions of Equations (33)–(35) with the parameters listed in the first line of Table 1. Coefficients $\alpha_{0}$, $\alpha_{1}$, $A_{1}$ and $B_{1}$ were calculated with the least-squares technique. Parameters *m*, *n*, and *a* were determined with the nonlinear regression method.

The accuracy of the fitting observations by means of Equations (38), (40) and (41) was assessed by means of the normalized root-mean-square deviation *R*. For coefficient *B*, for example, parameter $R_{B}$ was calculated with the following formula:

$$R_{B}=\frac{1}{\bar{B}}\sqrt{\frac{1}{M}\sum_{m=1}^{M}{\left(B_{m}-{\hat{B}}_{m}\right)}^{2}},$$

where *M* is the number of experimental points, $B_{m}$ is the value of *B* found in the *m*th test, ${\hat{B}}_{m}$ is its estimate by Equation (38), and

$$\bar{B}=\frac{1}{M}\sum_{m=1}^{M}B_{m}$$

is the means value of *B*. The normalized root-mean-square deviations $R_{A}$ and $R_{\alpha }$ for parameters *A* and α were calculated with the same procedure, with the help of Equations (40) and (41), respectively. Numerical analysis shows that the normalized root-mean-square deviations for coefficients α and *A* adopted the very low values of $R_{\alpha }=0.022$ and $R_{A}=0.073$. Parameter $R_{B}=0.173$ was slightly higher because practically all data in Figure 5C were close to zero.

**Figure 6:**
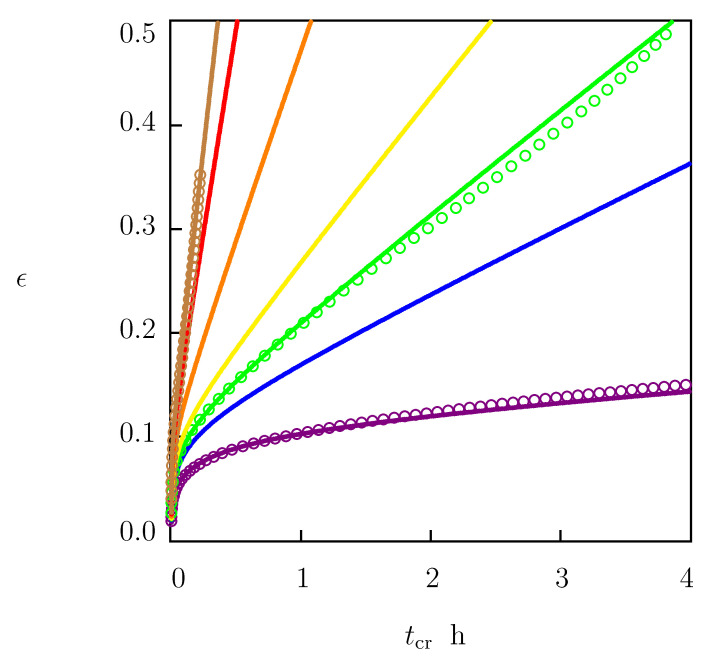
Tensile strain ϵ versus creep time $t_{\mathrm{cr}}$. Circles: experimental data in creep tests with stresses σ=15.0 (violet), σ=17.5 (green) and σ=20.5 (brown) MPa. Solid lines: predictions of the model for creep tests with various stresses σ: violet—15.0, blue—17.0, green—17.5, yellow—18.0, orange—19.0, red—20.0, brown—20.5 MPa.

To evaluate how accurately Equations (38), (40) and (41) describe the effect of stress σ on parameters α, *A* and *B*, we use these relations to predict the response of HDPE in tensile creep tests with stresses σ belonging to the interval between 15 and 21 MPa. For this purpose, the governing equations were solved numerically with the parameters reported in Table 1 and Table 2. Simulation results are presented in Figure 6, where predictions of the model are compared with the experimental data in tests with σ=15.0, 17.5 and 20.5 MPa (these data were used to find the coefficients collected in Table 2). Figure 6 shows reasonable agreement between the observations and their prediction by the model.

### 4.2. Validation of the Model

To examine the predictive ability of the model, two series of creep tests were performed on HDPE, and experimental creep diagrams were compared with the predictions of the model.

**Figure 7:**
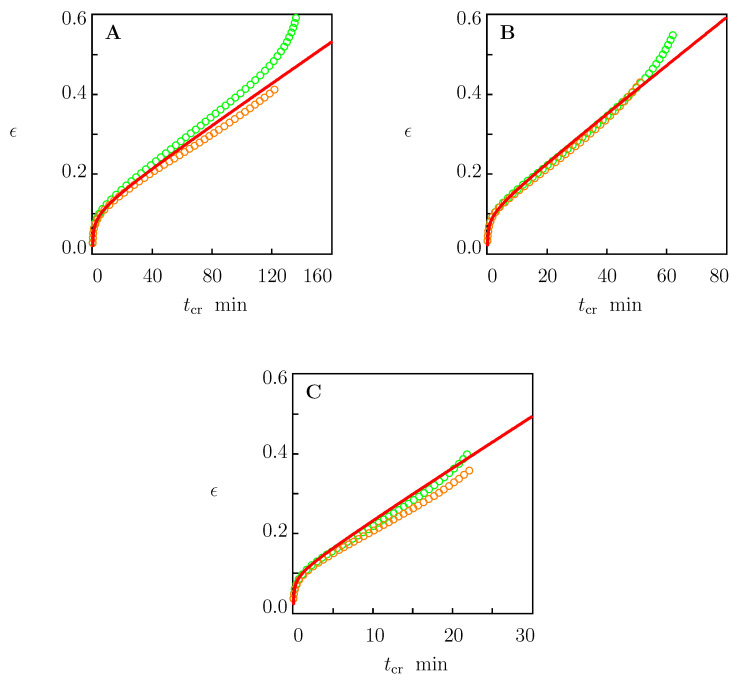
Tensile strain ϵ versus creep time $t_{\mathrm{cr}}$. Circles: experimental data in creep tests with tensile stresses σ on two specimens. Solid lines: predictions of the model. (**A**)—σ=18, (**B**)—σ=19, (**C**)—σ=20 MPa.

The first series of creep experiments (whose durations did not exceed 3 h) involved three tensile creep tests with stresses of σ=18, 19, and 20 MPa. In each test, a sample was loaded with the cross-head speed $\dot{d}=50$ mm/min until stress σ had reached its required value. Afterwards, the stress remained fixed, and an increase in strain ϵ was measured. The tests proceeded up to breakage of samples. Each test was repeated twice on different samples. Observations in these experiments are depicted in Figure 7 together with the results of simulation with the material parameters reported in Table 1 (the first line) and Table 2.

**Figure 8:**
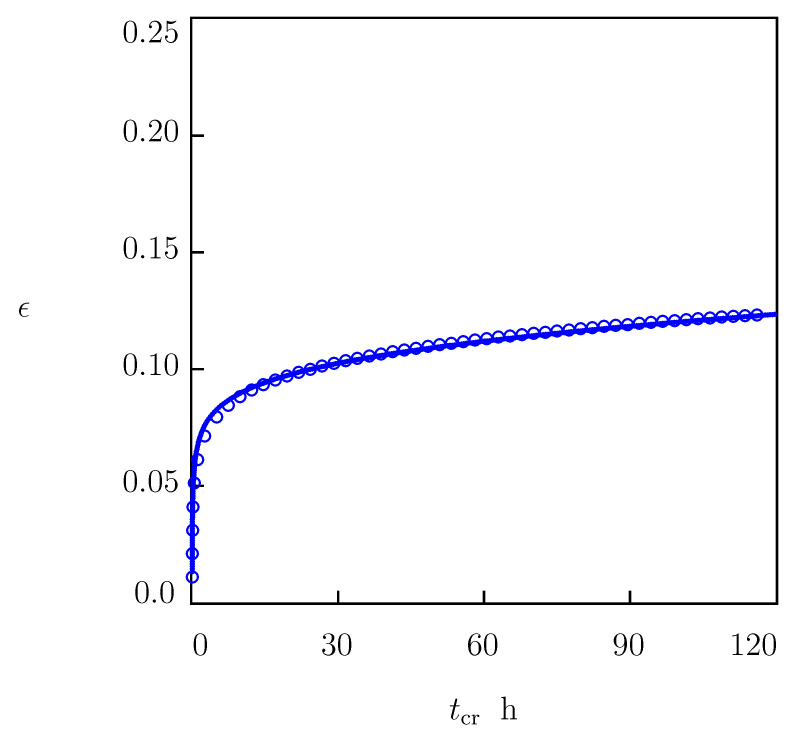
Tensile strain ϵ versus creep time $t_{\mathrm{cr}}$. Circles: experimental data in a creep test with tensile stress σ=12.0 MPa. Solid line: predictions of the model.

The other series consisted of a medium-term tensile creep test with stress of σ=12 MPa and duration of 120 h. The experimental creep diagram is depicted in Figure 8 together with predictions of the model with the parameters listed in Table 1 (the first line) and Table 2.

Figure 7 and Figure 8 confirm the ability of the model to predict the viscoelastoplastic behavior of HDPE in independent short- and medium-term creep tests.

### 4.3. Stress-Time-to-Failure Diagrams

To predict the time to failure $t_{f}$ of HDPE under creep conditions, we presumed samples to break when tensile strain ϵ reaches its ultimate value $\epsilon_{b}$. Given a tensile stress σ, the governing equations (with the material parameters listed in the first line of Table 1 and Table 2) were integrated numerically over time *t*. The approach was based on the assumption that the duration of the interval of tertiary creep was small compared with the intervals of primary and secondary creeps. This hypothesis was confirmed with the experimental data depicted in Figure 3C,D, Figure 4C and Figure 7.

Time to failure $t_{f}$ was determined from the maximal strain criterion [1]

(45) $$\epsilon \left(t_{f}\right)=\epsilon_{b},$$

where the strain at break $\epsilon_{b}$ was found from the tensile diagrams depicted in Figure 1.

**Figure 9:**
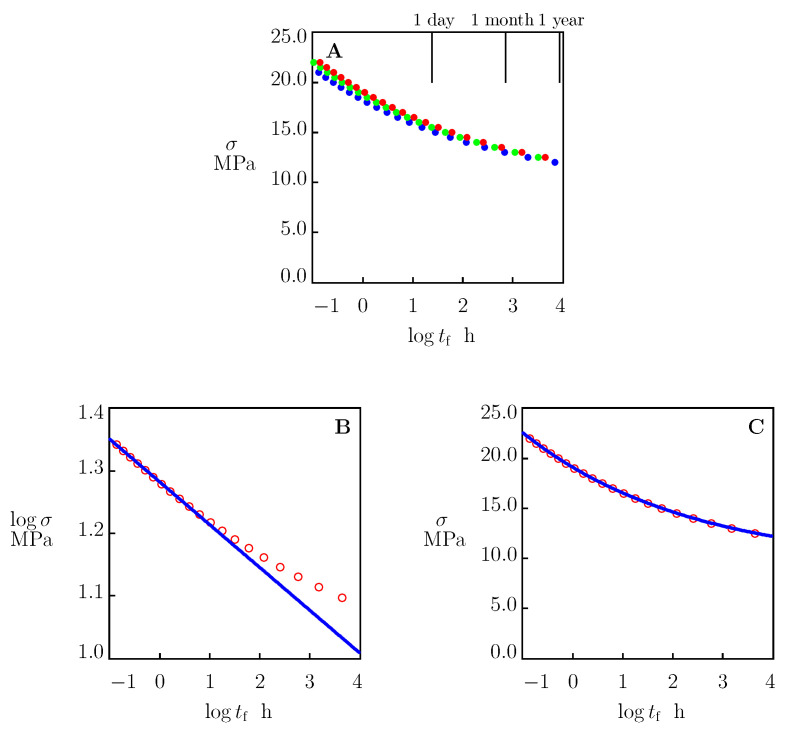
Tensile stress σ versus time-to-failure $t_{f}$. (**A**) Circles: predictions of the model with $\epsilon_{\mathrm{cr}}=0.3$ (blue), $\epsilon_{\mathrm{cr}}=0.4$ (green) and $\epsilon_{\mathrm{cr}}=0.5$ (red). (**B**,**C**) Circles: predictions of the model with $\epsilon_{\mathrm{cr}}=0.5$. Solid lines: their approximations with (**B**) the Eyring equation and (**C**) the power-law equation.

Figure 1 shows that, at all strain rates under consideration, the values of $\epsilon_{b}$ varied in a rather wide interval between 0.3 and 0.5. To assess how $t_{f}$ was affected by the width of this interval, a simulation was conducted of the governing equations under tensile creep conditions with $\epsilon_{b}=0.3$, 0.4, and 0.5. For each σ, time to failure $t_{f}$ was found from Equation (45). Stress σ is depicted as a function of $\log t_{f}$ in Figure 9A.

This figure shows that σ grew with $\epsilon_{b}$ when samples broke in the short-term creep tests with durations $t_{f}$ below 1 day. However, this effect became insignificant when the duration of tests exceeded 1 year. An important conclusion from Figure 9A is that uncertainties in the determination of strain at break $\epsilon_{b}$ do not affect the predictions of the model in long-term creep tests.

The stress-time-to-failure diagrams on solid polymers are conventionally described by means of the Eyring equation:

(46) $$\log\sigma =\sigma_{0}-\sigma_{1}\log t_{f},$$

where $\sigma_{0}$ and $\sigma_{1}$ are adjustable coefficients. A shortcoming of Equation (46) is that it does not take into account the creep endurance limit $\sigma_{*}$.

To assess at which times $t_{f}$ the presence of the endurance limit may affect the stress-time-to-failure diagram on HDPE, the governing equations were solved numerically with the material parameters listed in Table 1 and Table 2. Parameter $t_{f}$ was determined from Equation (45) with $\epsilon_{b}=0.5$. The results of simulation are presented in Figure 9B together with an approximation of the initial part of the stress-time-to-failure curve by Equation (46). According to this figure, the account for $\sigma_{*}$ induced noticeable deviations between results of simulation and prediction of Equation (46) even in medium-term creep tests (with $t_{f}$ exceeding 1 day).

Equation (46) is not the only relation used to predict the lifetime of polymers under creep conditions. The power-law equation

(47) $$\sigma =\sigma_{0}+\sigma_{1}{\left(\frac{t_{f0}}{t_{f}}\right)}^{\delta }$$

with $t_{f0}=1$ h and three material constants, $\sigma_{0}$, $\sigma_{1}$ and δ, provided an alternative for the Eyring formula. Equation (47) was derived in [1,49,50] on the basis of different scenarios for creep fracture. The stress-time-to-failure diagram reported in Figure 9B was replotted in Figure 9C together with its approximation by Equation (47) with the material parameters listed in Table 3. This figure shows that Equation (47) adequately predicts the time to failure $t_{f}$ in creep tests with durations up to several years.

It is conventionally presumed that the stress-time-to-failure curves for polyethylene and polyethylene pipes involve three intervals [7]. Equation (46) describes the lifetime along the first interval only (this interval corresponds to ductile failure of semicrystalline polymers). The other intervals are associated with quasibrittle failure driven by the slow growth of microcracks [51,52,53], and brittle failure caused by aging and chemical degradation [24,54,55]. Although the proposed model does not predict the lifetime of HDPE at the stages of semibrittle and brittle fracture, it improves predictions at the stage of ductile failure, and allows for the knee point corresponding to the transition from ductile to quasibrittle failure to be evaluated correctly.

## 5. Conclusions

A simple model was developed for the viscoelastic and viscoplastic behavior of semicrystalline polymers under arbitrary three-dimensional deformation with small strains. A polymer was treated as an equivalent network of chains bridged by permanent and transient bonds. The viscoplastic flow was associated with the sliding of junctions between chains with respect to their initial positions. Two mechanisms of slippage were taken into account that reflected the sliding of entanglements and tie chains in the amorphous regions, and the fine and coarse slips of lamellar blocks in spherulites. A novelty of our approach consists in the introduction of Equation (43) with the conformable fractional derivative to describe viscoplastic flow in the amorphous phase. An advantage of the model is that it allows for observations in mechanical tests to be described with the help of only seven material parameters (Figure 2 and Figure 3).

The experimental study involved tensile tests with various strain rates, tensile relaxation tests with various strains, and tensile creep tests with various stresses on injection-molded HDPE samples. It is revealed that the viscoelastic response in relaxation tests was independent of strains (Table 1). Simple phenomenological relations, namely, Equations (38), (40) and (41), were suggested to describe the effect of stress σ on coefficients α, *A*, and *B* characterizing the viscoplastic response. Coefficients in these equations are shown in Table 2.

The predictive ability of the model is demonstrated in Figure 7 and Figure 8. These figures show good agreement between results of numerical simulation and experimental data in independent short- and medium-term (up to 4 days) creep tests.

The model was applied to the analysis of stress-time-to-failure diagrams on HDPE under tensile creep. The following conclusions were drawn: (I) Although strains at break in the tensile tests $\epsilon_{b}$ varied in a rather wide interval (from 0.3 to 0.5), this uncertainty did not affect the accuracy of lifetime predictions in long-term tests whose durations exceed 1 year (Figure 9A). (II) Predictions of the model deviated from those based on the conventional Eyring relation when the durations of creep tests exceeded 1 day (Figure 9B). A reason for these discrepancies is that the proposed model takes into account the creep endurance limit (the stress below which the viscoplastic flow does not arise and the response is merely viscoelastic), while this parameter is disregarded in the conventional approach. (III) Predictions of the model were in good agreement with those based on the power-law relation (Figure 9C). The application of Equation (47) (instead of Equation (46)) allowed for a knee point on the failure diagram (characterizing transition from ductile to quasibrittle failure) to be determined.

## Figures and Tables

**Table 1:** Material parameters for the viscoelastic response.

ϵ	$\sigma_{0}$ MPa	γ s	Σ	κ
0.01	9.52	2.1	7.2	0.862
0.02	16.87	1.9	6.8	0.799
0.05	22.12	1.6	7.1	0.780

**Table 2:** Material parameters for the viscoplastic response.

Coefficient			
α	$\alpha_{0}$	$\alpha_{1}$	*m*
	1.98	$8.08\times 10^{-7}$	7.0
*A*		$A_{1}$	*a*
		$4.73\times 10^{-2}$	0.047
*B*		$B_{1}$	*n*
		$3.51\times 10^{-9}$	8.0

**Table 3:** Material parameters in Equation (47).

$\sigma_{0}$ MPa	$\sigma_{1}$ MPa	δ
9.21	9.88	0.3

## Data Availability

All data generated or analyzed during this study are included in the article.

## References

[1] Guedes R.M. (2006). Lifetime predictions of polymer matrix composites under constant or monotonic load. Compos. A.

[2] Chudnovsky A., Zhou Z., Zhang H., Sehanobish K. (2012). Lifetime assessment of engineering thermoplastics. Int. J. Eng. Sci..

[3] Laine E., Bouvy C., Grandidier J.-C., Vaes G. (2019). Methodology of accelerated characterization for long-term creep prediction of polymer structures to ensure their service life. Polym. Test..

[4] Sattar M., Othman A.R., Kamaruddin S., Akhtar M., Khan R. (2022). Limitations on the computational analysis of creep failure models: A review. Eng. Fail. Anal..

[5] Hutar P., Sevcik M., Nahlik L., Pinter G., Frank A., Mitev I. (2011). A numerical methodology for lifetime estimation of HDPE pressure pipes. Eng. Fract. Mech..

[6] Majid F., Elghorba M. (2017). HDPE pipes failure analysis and damage modeling. Eng. Fail. Anal..

[7] Zha S., Lan H.-q., Huang H. (2022). Review on lifetime predictions of polyethylene pipes: Limitations and trends. Int. J. Press. Vessel Pip..

[8] Nikolov S., Doghri I. (2000). A micro/macro-constitutive model for the small deformation behavior of polyethylene. Polymer.

[9] Drozdov A.D., Yuan Q. (2003). The viscoelastic and viscoplastic behavior of low-density polyethylene. Int. J. Solids Struct..

[10] Drozdov A.D., Gupta R.K. (2003). Constitutive equations in finite viscoelasticity of semicrystalline polymers. Int. J. Solids Struct..

[11] Dusunceli N., Colak O.U. (2006). High density polyethylene (HDPE): Experiments and modeling. Mech. Time-Depend. Mater..

[12] Drozdov A.D., de Christiansen J.C. (2007). Cyclic viscoplasticity of high-density polyethylene: Experiments and modeling. Comput. Mater. Sci..

[13] Drozdov A.D., de Christiansen J.C. (2007). Viscoelasticity and viscoplasticity of semicrystalline polymers: Structure–property relations for high-density polyethylene. Comput. Mater. Sci..

[14] Brusselle-Dupend N., Cangemi L. (2008). A two-phase model for the mechanical behaviour of semicrystalline polymers. Part I: Large strains multiaxial validation on HDPE. Mech. Mater..

[15] Drozdov A.D., de Christiansen J.C. (2008). Thermo-viscoelastic and viscoplastic behavior of high-density polyethylene. Int. J. Solids Struct..

[16] Ayoub G., Zairi F., Nait-Abdelaziz M., Gloaguen J.M. (2010). Modelling large deformation behaviour under loading-unloading of semicrystalline polymers: Application to a high density polyethylene. Int. J. Plast..

[17] Drozdov A.D. (2010). Cyclic thermo-viscoplasticity of high density polyethylene. Int. J. Solids Struct..

[18] Ayoub G., Zairi F., Frederix C., Gloaguen J.M., Nait-Abdelaziz M., Seguela R., Lefebvre J.M. (2011). Effects of crystal content on the mechanical behaviour of polyethylene under finite strains: Experiments and constitutive modelling. Int. J. Plast..

[19] Tripathi A., Mantell S., Le J.-L. (2019). A morphology based constitutive model for high density polyethylene. Mech. Mater..

[20] Amjadi M., Fatemi A. (2020). Creep and fatigue behaviors of high-density polyethylene (HDPE): Effects of temperature, mean stress, frequency, and processing technique. Int. J. Fatigue.

[21] Ayoub G., Rodriguez A.K., Mansoor B., Colin X. (2020). Modeling the visco-hyperelastic-viscoplastic behavior of photodegraded semi-crystalline low-density polyethylene films. Int. J. Solids Struct..

[22] Yan Z., Guo Q., Zairi F., Zaoui A., Jiang Q., Liu X. (2021). Continuum-based modeling large-strain plastic deformation of semi-crystalline polyethylene systems: Implication of texturing and amorphicity. Mech. Mater..

[23] Zhang Y., Ben Jar P.-Y., Xue S., Li L. (2019). Quantification of strain-induced damage in semi-crystalline polymers: A review. J. Mater. Sci..

[24] Hsueh H.-C., Kim J.H., Orski S., Fairbrother A., Jacobs D., Perry L., Hunston D., White C., Sung L. (2020). Micro and macroscopic mechanical behaviors of high-density polyethylene under UV irradiation and temperature. Polym. Degrad. Stab..

[25] Van Erp T.B., Reynolds C.T., Peijs T., Van Dommelen J.A.W., Govaert L.E. (2009). Prediction of yield and long-term failure of oriented polypropylene: Kinetics and anisotropy. J. Polym. Sci. Part B Polym. Phys..

[26] Carneiro Neto R.M., Akhavan-Safar A., Sampaio E.M., Assis J.T., da Silva L.F.M. (2022). Assessment of the creep life of adhesively bonded joints using the end notched flexure samples. Eng. Fail. Anal..

[27] Crissman J.M. (1991). On the long time creep and lifetime behavior in uniaxial extension of a linear low density polyethylene. Polym. Eng. Sci..

[28] Sedighiamiri A., Govaert L.E., Kanters M.J.W., van Dommelen J.A.W. (2012). Micromechanics of semicrystalline polymers: Yield kinetics and long-term failure. J. Polym. Sci. Part B Polym. Phys..

[29] Jar P.-Y.B. (2021). Revisiting creep test on polyethylene pipe—Data analysis and deformation mechanisms. Polym. Eng. Sci..

[30] Amjadi M., Fatemi A. (2020). Tensile behavior of high-density polyethylene including the effects of processing technique, thickness, temperature, and strain rate. Polymers.

[31] Zhu T., Li X., Zhao X., Zhang X., Lu Y., Zhang L. (2022). Stress-strain behavior and corresponding crystalline structures of four types of polyethylene under a wide range of strain rates. Polym. Test..

[32] Drozdov A.D., de Christiansen J.C., Klitkou R., Potarniche C.G. (2010). Viscoelasticity and viscoplasticity of polypropylene/polyethylene blends. Int. J. Solids Struct..

[33] Hiss R., Hobeika S., Lynn C., Strobl G. (1999). Network stretching, slip processes, and fragmentation of crystallites during uniaxial drawing of polyethylene and related copolymers. A comparative study. Macromolecules.

[34] Galeski A., Bartczak Z., Argon A.S., Cohen R.E. (1992). Morphological alterations during texture-producing plastic plane strain compression of high-density polyethylene. Macromolecules.

[35] Hong K., Rastogi A., Strobl G. (2004). A model treating tensile deformation of semicrystalline polymers: Quasi-static stress-strain relationship and viscous stress determined for a sample of polyethylene. Macromolecules.

[36] Drozdov A.D., Gupta R.K. (2003). Nonlinear viscoelasticity and viscoplasticity of isotactic polypropylene. Int. J. Eng. Sci..

[37] Okereke M.I., Akpoyomare A.I. (2019). Two-process constitutive model for semicrystalline polymers across a wide range of strain rates. Polymer.

[38] Tanaka F., Edwards S.F. (1992). Viscoelastic properties of physically cross-linked networks. Transient network theory. Macromolecules.

[39] Drozdov A.D., Klitkou R., de Christiansen J.C. (2013). Cyclic viscoplasticity of semicrystalline polymers with finite deformations. Mech. Mater..

[40] Naumenko K., Altenbach H., Gorash Y. (2009). Creep analysis with a stress range dependent constitutive model. Arch. Appl. Mech..

[41] Derrida B. (1980). Random-energy model: Limit of a family of disordered models. Phys. Rev. Lett..

[42] Yao H.-T., Xuan F.-Z., Wang Z., Tu S.-T. (2007). A review of creep analysis and design under multi-axial stress states. Nucl. Eng. Des..

[43] Williams S.J., Bache M.R., Wilshire B. (2010). Recent developments in analysis of high temperature creep and creep fracture behaviour. Mater. Sci. Technol..

[44] Wen J.-F., Tu S.-T., Xuan F.-Z., Zhang X.-W., Gao X.-L. (2016). Effects of stress level and stress state on creep ductility: Evaluation of different models. J. Mater. Sci. Technol..

[45] Khalil R., Horani M.A., Yousef A., Sababheh M. (2014). A new definition of fractional derivative. J. Comput. Appl. Math..

[46] Abdeljawad T. (2015). On conformable fractional calculus. J. Comput. Appl. Math..

[47] Drozdov A.D. (1997). Fractional differential models in finite viscoelasticity. Acta Mech..

[48] Sumelka W. (2014). Fractional viscoplasticity. Mech. Res. Comm..

[49] Schapery R.A. (1975). Theory of crack initiation and growth in viscoelastic media. 3. Analysis of continuous growth. Int. J. Fract..

[50] Bi G., Li Z., Wang Q., Jiang D. (2022). An examination of creep failure criterion based on a strain threshold identified with a power law model. Mech. Time-Depend. Mater..

[51] Frank A., Arbeiter F.J., Berger I.J., Hutar P., Nahlik L., Pinter G. (2019). Fracture mechanics lifetime prediction of polyethylene pipes. J. Pipeline Syst. Eng. Pract..

[52] Wee J.-W., Park S.-Y., Choi B.-H. (2020). Modeling and application of discontinuous slow crack growth behaviors of high-density polyethylene pipe with various geometries and loading conditions. Eng. Fract. Mech..

[53] Wee J.-W., Chudnovsky A., Choi B.-H. (2022). Modeling of multiple crack initiation in polymer pipes under oxidative environment. Int. J. Eng. Sci..

[54] Ojeda T., Freitas A., Birck K., Dalmolin E., Jacques R., Bento F., Camargo F. (2011). Degradability of linear polyolefins under natural weathering. Polym. Degrad. Stabil..

[55] Grause G., Chien M.-F., Inoue C. (2020). Changes during the weathering of polyolefins. Polym. Degrad. Stabil..

